# Characteristics of the Epididymal Luminal Environment Responsible for Sperm Maturation and Storage

**DOI:** 10.3389/fendo.2018.00059

**Published:** 2018-02-28

**Authors:** Wei Zhou, Geoffry N. De Iuliis, Matthew D. Dun, Brett Nixon

**Affiliations:** ^1^Priority Research Centre for Reproductive Science, School of Environmental and Life Sciences, University of Newcastle, Callaghan, NSW, Australia; ^2^Faculty of Health and Medicine, University of Newcastle, Callaghan, NSW, Australia; ^3^Cancer Research Program, School of Biomedical Sciences and Pharmacy, Hunter Medical Research Institute, University of Newcastle, New Lambton Heights, NSW, Australia

**Keywords:** epididymis, sperm maturation, intracellular communication, protein trafficking, apocrine secretion, merocrine secretion, epididymosome, dynamin

## Abstract

The testicular spermatozoa of all mammalian species are considered functionally immature owing to their inability to swim in a progressive manner and engage in productive interactions with the cumulus–oocyte complex. The ability to express these key functional attributes develops progressively during the cells’ descent through the epididymis, a highly specialized ductal system that forms an integral part of the male reproductive tract. The functional maturation of the spermatozoon is achieved *via* continuous interactions with the epididymal luminal microenvironment and remarkably, occurs in the complete absence of *de novo* gene transcription or protein translation. Compositional analysis of the luminal fluids collected from the epididymis of a variety of species has revealed the complexity of this milieu, with a diversity of inorganic ions, proteins, and small non-coding RNA transcripts having been identified to date. Notably, both the quantitative and qualitative profile of each of these different luminal elements display substantial segment-to-segment variation, which in turn contribute to the regionalized functionality of this long tubule. Thus, spermatozoa acquire functional maturity in the proximal segments before being stored in a quiescent state in the distal segment in preparation for ejaculation. Such marked division of labor is achieved *via* the combined secretory and absorptive activity of the epithelial cells lining each segment. Here, we review our current understanding of the molecular mechanisms that exert influence over the unique intraluminal environment of the epididymis, with a particular focus on vesicle-dependent mechanisms that facilitate intercellular communication between the epididymal soma and maturing sperm cell population.

## Introduction

**Figure 1:**
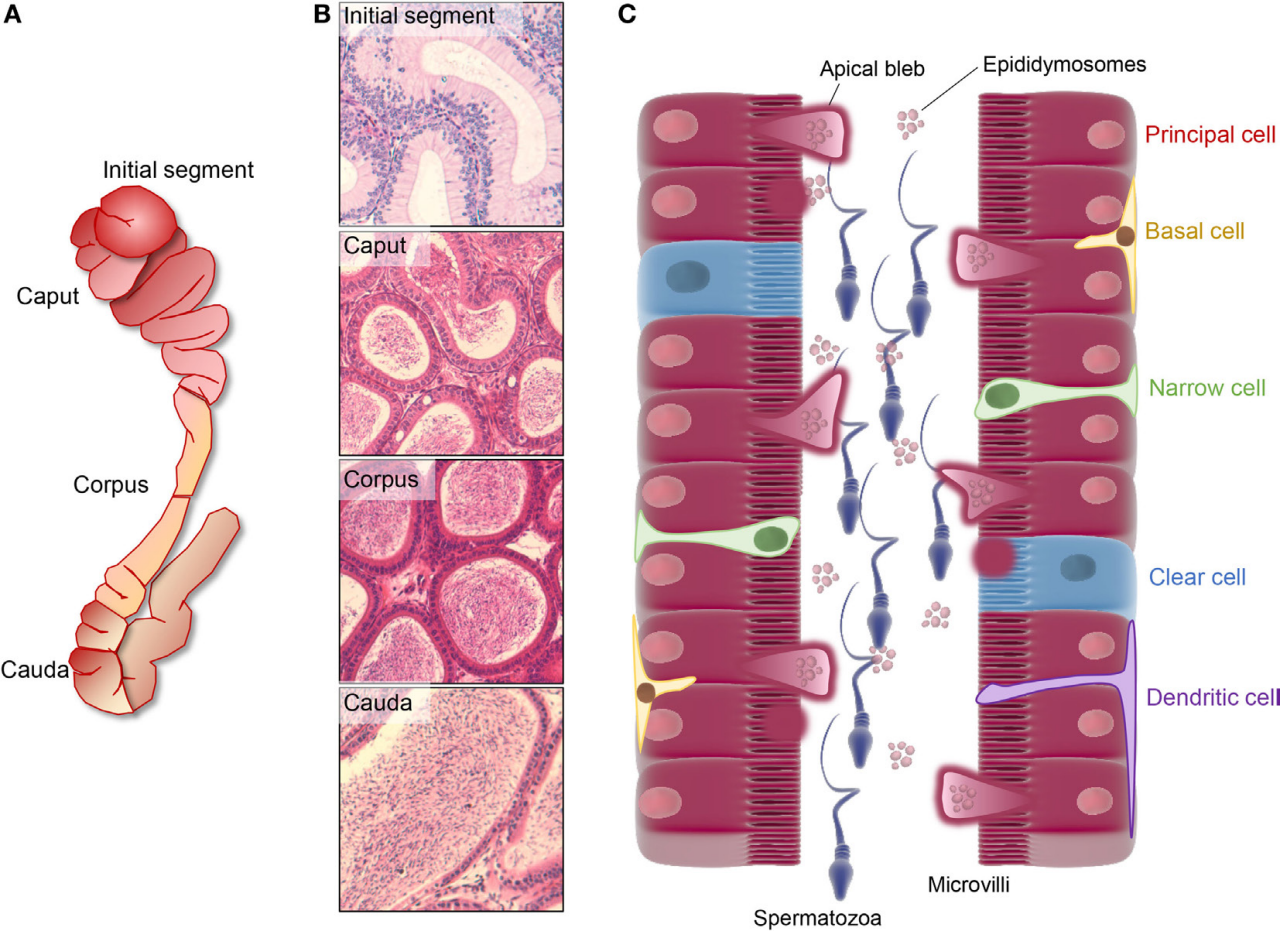
Regionalized structure and schematic distribution of the major cell types in the mouse epididymis. **(A,B)** The mouse epididymis is generally broadly divided into four unique anatomical segments: the initial segment, the caput, corpus, and cauda epididymis. The initial segment is a loosely coiled tubule with a wide diameter and a low concentration of spermatozoa. Epithelial cells in this segment are elongated and possess high stereocilia. The caput segment is characterized by a narrow luminal diameter, while both the luminal diameter and the sperm concentration increase distally within the corpus and cauda epididymis. Differing cell types within these segments are responsible for the creation of a specialized luminal microenvironment that promotes the sequential maturation of spermatozoa (caput and corpus epididymis) and their subsequent storage (cauda epididymis). **(C)** Principal cells dominate the soma along the entire length of epididymis and are particularly active in terms of protein biosynthesis and secretion in the proximal epididymal segments. In this context, an apocrine pathway of secretion, featuring the formation and eventual shedding of large bleb-like structures from the apical margin (i.e., apical blebs) of principal cells, appears to be a dominant secretory mechanism operating in all epididymal segments. Upon degradation within the epididymal lumen, apical blebs release a heterogeneous population of membranous extracellular vesicles, termed epididymosomes, which have been implicated in intracellular communication with spermatozoa and downstream epithelial cells. Aside from principal cells, clear cells are distributed sporadically throughout the epithelium of the caput, corpus, and cauda segments in most studied species and are primarily responsible for selective absorption of luminal components and conversely, the regulation the luminal pH. A suite of additional cell types have been described in the epididymis, including basal cells, apical cells, halo cells, narrow cells (only found in initial segment and intermediate zone), and immunological cells.

The mammalian epididymis is an exceptionally long, convoluted ductal system that serves to connect the ductuli efferentes, which drain the testes, to the ductus deferens. Anatomically, this highly specialized organ is generally divided into four broad segments: the initial segment, caput, corpus, and cauda epididymides (Figures 1A,B) (1); although this demarcation is not strictly adhered to in all mammalian species (2). Irrespective, the epididymis is responsible for the provision of an optimal environment to promote the functional transformation of spermatozoa and their subsequent storage in viable state in readiness for ejaculation. Functional profiling studies indicate that the epididymis displays highly regionalized characteristics. Thus, the initial segment and upstream ductuli efferentes are responsible for the absorption of the majority of testicular fluid entering the duct leading to a pronounced concentration of the luminal spermatozoa (3). Thereafter, the caput epididymis is most active in terms of protein synthesis and secretion, and a small portion of the sperm passing through this region begin to exhibit the ability to swim in a progressive manner and to recognize an oocyte (4–6). These functional characteristics continue to develop in the corpus epididymis before reaching an optimal level in the distal caudal segment. This latter region is characterized by a relatively large lumen and its surrounding epithelial cells exhibit strong absorptive activity (7, 8). Such attributes align with the dominant function of the cauda epididymis in terms of the formation of a sperm storage reservoir.

It is well established that the combined secretory and absorptive activities of the epididymal epithelial cells are responsible for the creation of the highly specialized luminal microenvironment that promotes the gradient of increasing fertility in the sperm population held therein (9). Systematic analysis of the composition of these luminal fluids has revealed a complex macromolecular landscape encompassing a myriad of soluble factors in addition to non-pathological amyloid matrices and exosome-like vesicles termed epididymosomes. The latter of these have come under increasing scrutiny owing to their potential to facilitate the efficient transfer of a variety of protein and small non-coding RNA cargo to the maturing sperm cells. Furthermore, there is emerging evidence that the epididymosome payload may be dynamically altered in response to paternal exposure to environment stressors. The implications of such changes in terms of establishing the sperm epigenetic and proteomic signatures, and their potential to influence the downstream health and developmental trajectory of offspring, are only just beginning to be realized. It is therefore timely to review the changes associated with sperm maturation in the epididymal tract and the molecular mechanisms by which such changes are brought about. Here, we focus on the regulation of the epididymal luminal microenvironment and place particular emphasis on vesicle-dependent mechanisms that facilitate intercellular communication between the lining epididymal soma and the maturing sperm cell population.

## The Luminal Microenvironment of the Epididymis

The process of post-testicular sperm maturation is reliant on the highly specialized intraluminal microenvironment of the epididymis, arguable one of the most complex milieus produced by any endocrine gland. Accordingly, the selective ablation of genes that lead to dysregulation of the epididymal microenvironment commonly results in male infertility/subfertility phenotypes (Table 1). The origin of these fluids rests with the pseudostratified epithelium lining the duct. This epithelium comprises a number of different cell types (Figure 1C) including, populations of principal, clear, narrow, apical, basal, halo, and immunological (macrophage and dendritic) cells; the abundance of which varies considerably between different epididymal segments (10). Detailed functional studies have confirmed spatial differences in the profile of each cell type and revealed that, under the precise control afforded by androgens and various other factors of testicular origin, they each make unique contributions to sperm maturation, protection, and storage (10).

**Table 1:** Gene knockout or deletion strategies that impact the intraluminal environment of the mouse epididymis.

Gene knockout	Fertility phenotype	Changes to epididymal environment	References (PMID)
*Sed1*	Infertile	Hypo-osmotic and alkaline epididymal fluid, disrupted fluid reabsorption, increased intracellular vesicles	20122713
*Esr1*	Infertile	Hypo-osmotic fluid	20130266
*C-ros*	Infertile	Defective initial segment development, increased luminal pH	10645273, 15095336
*Dicer1*	Subfertile	Imbalanced lipid homeostasis in proximal segments. Dedifferentiation of the epithelium and imbalance in sex steroid signaling	25366345, 22701646
*Rlx*	Subfertile	Delayed maturation and growth associated with increased collagen deposition	15956703
*He6*	Infertile	Reduced in size and dysregulation of fluid reabsorption	15367682
*Lur (testosterone treatment)*	Subfertile	Inflammation in epididymis	15514086
*Erα*	Infertile	Disruption in Na^+^ reabsorption and passive water transport, abnormal epithelial ultrastructure	11698654
*Nhe3*	Infertile	Disruption in Na^+^ reabsorption and passive water transport	11698654
*Lxr*	Infertile	Abnormal accumulation neutral lipids	15525595
*Apoer2*	Infertile	Dysfunction of clusterin and PHGPx protein impacting sperm maturation	12695510
*Fsh-r*	Subfertile	Smaller epithelial surface area in caput and corpus segments	15973687
*Gpx5*	Higher incidence of miscarriages and developmental defects	Excess of reactive oxygen species in the cauda segment leading to oxidative damage of spermatozoa	19546506
*Hoxa10Hoxa11*	SubfertileInfertile	Epididymis characterized by homeotic transformationEpididymis characterized by homeotic transformation	87877437789268
*Hexa*	Infertile (age dependent)	Inability to degrade endocytosed substrates	12617783
*Tmf*	Infertile	Epithelial apoptosis and sperm stasis in the cauda segment	23000399
*Trpv6*	Subfertile	Defects in epididymal Ca^2+^ absorption	22427671
*Slc9a3*	Infertile	Abnormally abundant secretions and calcification in the lumen	28384194

### Epididymal Epithelium

Principal cells represent the major cell type throughout the entire epididymis, constituting as much as 80% of the peritubular interstitium (11). These cells are characterized by abundant secretory apparatus [endoplasmic reticulum (ER), Golgi and secretory granules] reflecting their high exocytotic activity, especially in the proximal portions of the epididymis (caput and corpus) (8, 12, 13). In more distal segments (cauda), the principal cells take on a predominantly endocytotic role in which they are actively responsible for the reabsorption of various components from the epididymal fluid, a function that is also shared with that of the clear cell population (12). Clear cells are the second most abundant cell type, being widely distributed in the caput and corpus segments but displaying most enrichment in the cauda. The apical domain of clear cells is replete with endocytotic apparatus and accordingly, these cells have a tremendous capacity for endocytosis (7, 12). This is particularly true of the clear cells that populate the distal epididymal segments, which have been implicated in the uptake and disposal of the cytoplasmic droplets that are shed from the maturing sperm cell population (7), as well as the recycling of various other luminal components. Also within their apical domain, clear cells possess key elements of the machinery [vacuolated (V)-ATPase, carbonic anhydrase II, and soluble adenylate cyclase] necessary for acidification of the luminal environment, thus highlighting their role in regulation of the pH of the epididymal environment (8, 14). Narrow and apical cells are mainly found within the initial segment (15, 16). However, their function is yet to be fully resolved (8). Basal cells display a hemispherical morphology and form intimate contact with the basement membrane and principal cells on both sides (8). Ligation experiments in the rat have shown that basal cells possess the ability to change shape in order to adjust the luminal volume and pressure; features that are suggestive of a protective role in preserving the structural integrity of the lumen (17).

The remaining cell types comprising the epididymal epithelium are predominantly related to immune functions (8), an important consideration given the highly antigenic nature of the male germ cell. Indeed, the epididymal luminal environment and thus maturing spermatozoa are shielded from immune surveillance behind a blood–epididymal barrier. This barrier consists of a network of tight junctions that form between adjacent epithelial cells. The ductal system so created is further characterized by connective septa to form a number of distinct segments and thus facilitate the formation of successive, regionally distinct luminal microenvironments (18–20). Since a majority of testicular fluid is reabsorbed before reaching the proximal segments of the epididymis, most of the luminal components, other than the sperm cells themselves, originate from the secretory activity of the pseudostratified epithelial cells comprising the duct. Detailed compositional analysis of the epididymal fluid has revealed it contains a complex array of proteins, ions, and small non-coding RNA species (21, 22). While these molecules are present in all the epididymal segments, they nonetheless display an extraordinary level of regionalization, which reflect differential secretory and absorptive activity of the epithelial cells.

### Luminal Components

As previously mentioned, the proteome of maturing spermatozoa is substantially modified *via* the uptake, repositioning, and posttranslational modification of a significant portion of proteins. Such changes are mediated by direct exposure to the proteins secreted into the epididymal luminal environment, with a majority of these originating in the proximal segments of the caput and corpus epididymis (23). By contrast, proteins involved in the preservation of sperm viability, such as antioxidant enzyme defenses, and those responsible for suppression of humoral immune responses, tend to be enriched in the cauda epididymal secretome (23). Furthermore, detailed transcriptomic analysis in the mouse epididymis has identified a prominent theme of segment-dependent regulation, with the expression of 12.8% of the total 17,000 epididymal genes being characterized by changes of at least fourfold between any two segments (21). This ratio increases to 35.8% if the criterion is relaxed to include transcripts that vary by at least twofold. Consistent with these data, it has also been shown that protein and gene expression patterns display very discrete profiles that closely align with the borders of septa demarcating anatomically different segments of the epididymis (24–26). How this precise regulation is imposed is still unclear, but it is perhaps notable that grossly similar profiles of epididymal protein expression have been documented along the epididymal tract of many mammalian species, including large domestic species (27) and humans (28). One possible explanation rests with evidence for the expression of a myriad of small non-protein coding RNA transcripts in the epithelial cells (22, 29–33).

In this context, the focus for most investigations has been the microRNA (miRNA) class of RNA molecules (~21–25 nucleotides) that hold a key regulatory role in the repression of mRNA translation. Indeed, the use of microarray and next generation sequencing methodologies has led to the identification of a total of 545 and 370 miRNAs in the human and mouse epididymis, respectively (22, 29). A large portion of these miRNAs are conserved among different epididymal segments (75% in the mouse epididymis) and even between different species (31% between mouse and human), suggesting housekeeping roles in the regulation of epididymal homeostasis (22). In contrast, other miRNAs are characterized by pronounced segmental patterns of expression (15% in mouse epididymis). For example, *miR-204-5p* and *miR-196b-5p* are down and upregulated significantly, with approximately 39- and 45-fold differences in expression having been recorded between caput and caudal segments of the mouse epididymis (22). The biological implications of such differences are highlighted by the potential for each miRNA species to exert regulatory control over multiple targets. By way of illustration, target prediction algorithms indicate that an estimated 530 and 160 genes are putatively able to be targeted by *miR-204-5p* and *miR-196b-5p*, respectively (34). Thus, the differing miRNA expression profiles documented in each epididymal segment impose a daunting level of complexity to the regulation of unique segmental environments in the epididymis. The precise mechanisms responsible for differential miRNA expression profiles remains poorly understood but are undoubtedly influenced by androgens and other lumicrine factors of testicular origin (35). Alternatively, it has been proposed that epididymosomes may serve as vectors to selectively traffic miRNA cargo between their sites of production in the proximal epididymal segments to recipient epithelial cells lining downstream segments (36).

In any case, it is therefore perhaps not surprising that intraluminal proteome of the epididymis ranks among the most complex produced by any endocrine gland. In addition to a diversity of soluble proteins, electron-dense proteinaceous complexes have also been described in the epididymal lumen of rodents, rams and, recently humans (37–40). These are apparently non-pathological structures, with a diameter ranging from 500 nm to 1.2 µm, which lack any obvious organelles, and similarly, are not delineated by a lipid bilayer (39). Despite these features, the formation/maintenance of these extracellular matrices appears to be a selective rather than stochastic process as evidenced by the conservation of their protein profile across several taxa. In this regard, the highly amyloidogenic cystatin-related epididymal spermatogenic (CRES) family appear to be critical constituents of these entities (41). Thus, a potential mechanism for formation is through self-assembly brought about by interaction of CRES subgroup members, small hydrophobic proteins and/or prion proteins (37, 41). Accordingly, pioneering work by the Cornwall laboratory has established that these extracellular matrices do possess amyloid structural properties, which change along the length of the epididymal tubule. Indeed, immature amyloid forms prevail in the proximal epididymis before taking on thinner “film-like” characteristics in the distal epididymis (41, 42). Notably, these changes in amyloid matrix structure parallel changes in epididymal function with proximal segments responsible for promoting sperm maturation and distal segments serving primarily as a storage site for mature spermatozoa (please see Introduction). On the basis of these data, it has been suggested that epididymal amyloids are formed for functional purposes in sperm maturation and/or protection by coordinating interactions between the luminal fluid and spermatozoa. Interestingly, a similar role has also been proposed for “dense bodies” that have been documented in the lumen of the rodent epididymis (39, 43, 44), although at present it remains to be established whether these amorphous entities do equate to amyloid matrices. Irrespective, dense bodies are replete with proteins such as those of the molecular chaperone family (HSPD1 and HSP90B1) (39), bactericidal/permeability-increasing protein (43), and glycogen synthase kinase 3. While the precise function of the chaperone cargo remains obscure, it has been suggested that, similar to CRES, these proteins may assist in the aggregation of luminal proteins into large discrete entities, and thus increase the efficiency with which they are able to be delivered to the spermatozoa. In keeping with this notion, ultrastructural analyses have provided evidence that dense bodies form intimate contact with epididymal spermatozoa, and thereafter mediate the transfer of associated cargo (44). Further dissection of the structural and functional properties of these extracellular matrices promises to shed new light on the mechanisms by which the epididymal soma communicates with sperm to coordinate their maturation and storage.

In addition to amyloids/dense bodies, the epididymal lumen also features an impressive population of extracellular vesicles. Indeed, as early as 1985, Yanagimachi identified a population of small membranous vesicles residing near the surface of epididymal spermatozoa in the Chinese hamster, and subsequently predicted their potential role in cholesterol transfer to the maturing spermatozoa (45). The existence of these vesicles, now commonly referred to as “epididymosomes,” has subsequently been confirmed in the epididymal fluid of a variety of other mammalian species such as mice (46), rats (47), bull (48, 49), and human (50). With defining characteristics of a relatively small size (varying from 50–500 nm), a heterogeneous cargo of macromolecules, and a membrane that is highly enriched in cholesterol (51), epididymosomes have since been implicated in promoting various aspects of sperm maturation. Such influence is mediated through either direct interaction with the sperm themselves or *via* indirect mechanisms involving delivery of regulatory cargo (e.g., miRNAs; see below) to epithelial cells downstream of their site of genesis.

Proteomic analysis of bull and human epididymosomes has revealed they contain a complex cargo of several hundred proteins encompassing key classes of enzymes, chaperones, structural proteins, and many more with hitherto unknown function (52, 53). Some of these proteins have been shown to be directly transferred to specific sperm domains during their transit through the epididymis and are, in turn, essential for promoting the functional maturity of these cells. Notable examples include macrophage migration inhibitory factor (MIF), a cytokine with a broad distribution and diverse functions in multiple tissues. During epididymal transit in the rat and bovine, MIF is transferred from epididymosomes to the fibrous sheath of the sperm flagellum and subsequently influences the motility characteristics of these cells (54, 55). Alternatively, P26h/P34H family members (P26h in hamster, P25b in bovine, and P34H in humans) are a group of glycosyl-phosphatidylinositol (GPI)-linked proteins that are initially found within epididymosomes before becoming firmly anchored to the surface of the sperm acrosomal domain. Functional studies have revealed that these proteins are indispensable for zona pellucida binding, which is a prerequisite for successful fertilization (56–58). Other transferred proteins include membrane-associated, transmembrane, and GPI-linked candidates, and it is likely that the epididymosomes afford an important mechanism for the bulk delivery of this cargo, possibly in the form of already assembled protein complexes to the sperm cell. Direct evidence for this form of transport has been provided through *in vitro* co-incubation studies between spermatozoa and epididymosomes focusing on protein complexes such as the MCA4a–PMCA4b–CASK complex, which has been directly co-immunoprecipitated from epididymosomes (59). This protein complex has been shown to be transferred to the acrosomal region and mid-piece of the flagellum (which are two key functional domains in the male gamete) under optimized *in vitro* incubation conditions featuring the physiologically relevant pH of 6.5 and a high concentration of zinc (60, 61).

In this sense, the ionic composition of epididymal fluids is markedly different from that documented in other bodily fluids (62). Thus, the epididymal fluid contains a lower overall concentration of Na^+^, Cl^−^, Ca^2+^ (except for human), and $HCO_{3}{\;}^{-}$ ions than those that have been reported in blood plasma (62–65). These particular ionic concentrations are, in turn, tightly associated with the regulation of luminal acidification that helps keep spermatozoa in a dormant state (66, 67). Within the epididymal lumen, events such as sexual arousal stimulate principal cells to secrete $HCO_{3}{\;}^{-}$ and Cl^−^. This change is sensed by purinergic receptors in adjacent clear cells where it leads to activation of bicarbonate-sensitive adenylyl cyclase and the downstream re-localization of proton pumping ATPases to the apical region of these cells. These pumps subsequently secrete H^+^ (66) leading to further acidification of the epididymal luminal environment. This cell–cell cross talk is mediated by several membrane receptors including cystic fibrosis conductance transmembrane regulator positioned in the principal cells and cognate sodium bicarbonate cotransporters (NBC) in the clear cells. Intracellular Ca^2+^ is also required for ATPase sequestration within the apical domain of clear cells owing to its ability to dynamically modulate the actin cytoskeleton (68). Ca^2+^ also plays a more direct role in the regulation of sperm functionality by virtue of its ability to enter the cell through cation channels (CATSPER) that are located in the principal piece of the sperm flagellum. Accordingly, blocking Ca^2+^ influx *via CatSper* knockout strategies leads to impaired sperm motility (69).

Finally, in addition to the more well-studied proteomic and ionic components of the epididymal luminal fluids, a number of recent studies have provided compelling evidence for the existence of myriad of small non-protein coding RNA transcripts in epididymal fluid (29–33). Such entities appear predominantly, but perhaps not exclusively, to be associated with epididymosomes (33, 70–72). In the mouse model, we have confirmed that epididymosomes encapsulate >350 different miRNAs. This inventory includes many miRNAs that are found in epididymosomes and epididymal spermatozoa but are apparently absent, or detected at significantly reduced levels, in the surrounding soma. Such findings are suggestive of selective packaging of the epididymosomes cargo (52). In keeping with this notion, substantial qualitative and quantitative changes have been documented in the epididymosome cargo along the length of the epididymis; including significant fold changes in the accumulation of almost half of their encapsulated miRNAs (70). These data accord with similar findings in the bull (33), and take on added significance in view of evidence that epididymosomes can convey their macromolecular payload to spermatozoa and downstream epididymal epithelial cells (33). Epididymosomes thus represent key conduits for the selective modification of the sperm proteome and epigenome during their post-testicular maturation (71, 73). A challenge for future studies will be to determine the extent to which this novel form of intercellular communication underpins the perturbation of the sperm epigenome arising in response to paternal environmental exposures (74).

## Regulation of the Epididymal Luminal Environment

Sequential modification of the epididymal luminal milieu demands the interchange of components between the lining epithelium and the lumen. As described previously, this form of intercellular communication is carefully orchestrated by the secretory and absorptive activity of the differing populations of epithelial cells (8). Logically, the interface for a majority of these interactions is the sperm plasma membrane, a structure that is known to undergo dramatic maturational changes mediated by either direct contact with the epithelial margin or by physical exchange of luminal components. Although such exchanges undoubtedly rely on membrane trafficking activity, the precise mechanisms and the machinery involved in these events remains to be fully elucidated.

### Epithelial Secretion: Merocrine versus Apocrine Secretory Pathways

**Figure 2:**
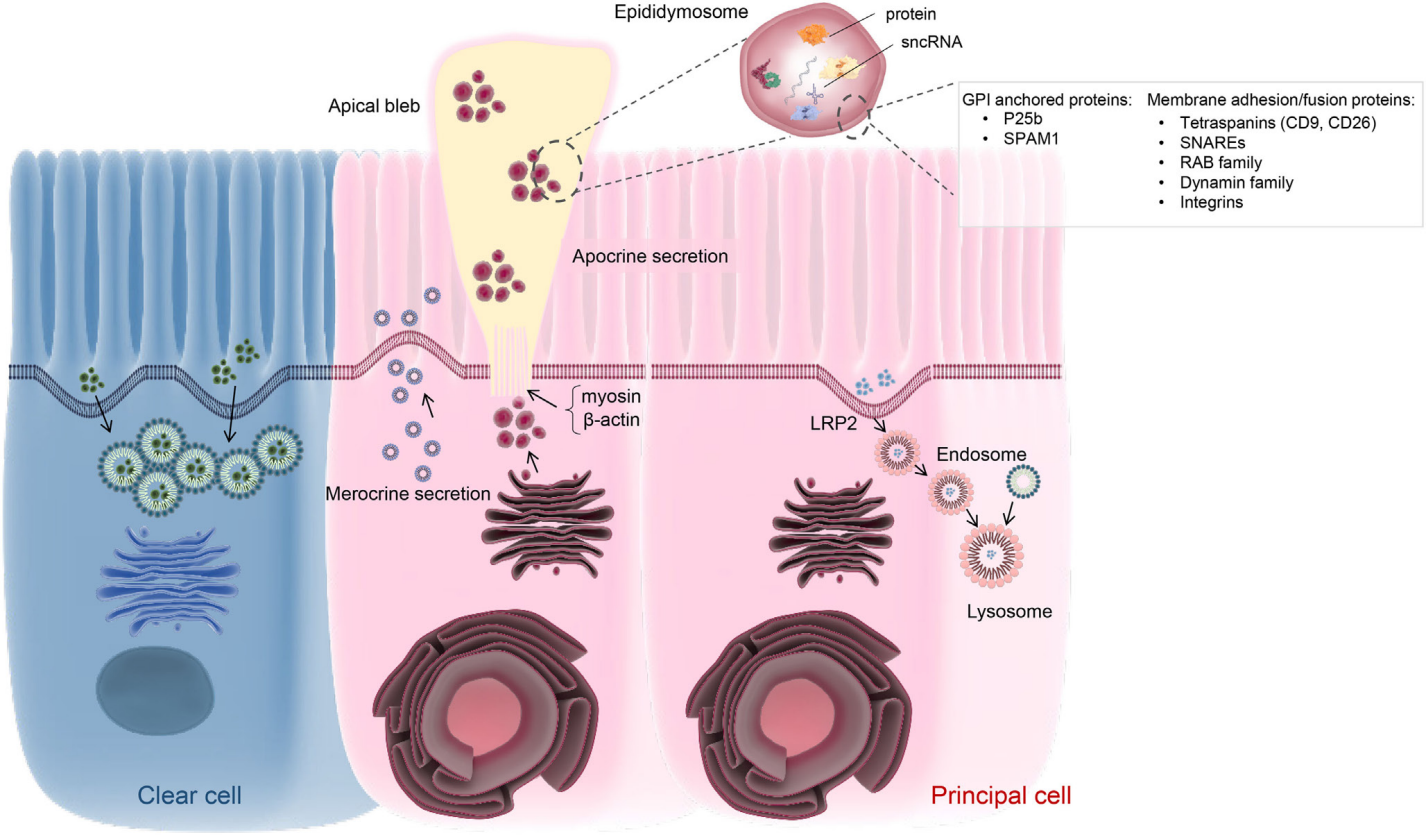
Schematic of vesicle-dependent mechanisms that contribute to the creation of the highly specialized epididymal intraluminal milieu. Principal cells represents the dominant cell type throughout the length of the epididymis and are particularly active in terms of protein biosynthesis and secretion. At least two active secretory pathways have been documented in these cells, namely classical merocrine secretion and an alternative apocrine secretory pathway. The former is characterized by secretory vesicles formed by the Golgi apparatus and leads to the release of a myriad of soluble proteins. In contrast, apocrine secretion provides a pathway for the secretion of glycosyl-phosphatidylinositol-linked proteins and other hydrophobic proteins. These proteins generally require posttranslational modification in the Golgi apparatus, prior to being selectively packaged into exosome-like vesicles referred to as epididymosomes. These small vesicles are sequestered into large bleb-like structures protruding from the apical margin of the epithelial cells. The attachment anchoring the blebs to their parent cell progressively narrows to form a stalk-like process (potentially through reorganization of cytoskeletal proteins such as myosin and β-actin) and eventually undergoes scission leading to their shedding into the lumen and eventual degradation. The epididymosomes so released provide a mechanism for intercellular communication, thereby enabling the delivery of a complex macromolecular payload to recipient cells in the form of luminal spermatozoa and/or downstream epithelial cells. Such cargo are known to include several hundred proteins as well as various species of small non-protein coding RNA (sncRNA). At present, the mechanisms by which epididymosomes are tethered, and deliver their cargo, to recipient cells remains to be equivocally determined although various proteins have been implicated in this process. Principal cells may also participate in endocytosis, involving the uptake of epididymal luminal contents via receptor (e.g., LRP2) mediated mechanism(s). A similar function has also been assigned to the clear cell population, which is mainly responsible for the recycling of luminal components.

Merocrine secretion is a classical pathway operative in glandular tissue whereby the endosomal network generates, packages, and finally exports cargo *via* exocytosis (Figure 2). The diverse proteins secreted in this fashion share the general properties of being soluble and containing a signal peptide sequence that directs them toward the ER in preparation for trafficking to the membrane (75). In the mammalian epididymis, merocrine secretion is believed to be one of the major pathways through which principal cells are able to regulate the composition of the intraluminal milieu. Accordingly, morphological characterization of this cell population has revealed they possess extremely long microvilli accompanied by numerous vesicles extending from the Golgi apparatus to the adluminal cell border; this is particularly true of the proximal caput segment, which is most active in terms of protein secretion into the lumen (76). Among the epididymal proteins secreted *via* the merocrine pathway, many have been implicated in forming loose electrostatic associations with the periphery of the sperm surface (77). This appears to be true of proteins such as those implicated in holding sperm in a decapacitated state, i.e., the so-called decapacitation factors (77).

The precise mechanisms controlling merocrine secretion are still relatively poorly understood. Some studies have implicated regulatory elements of classical membrane trafficking machinery, such as the Rab superfamily of monomeric GTPases (78). Although various Rab proteins are highly, and differentially, expressed in the epididymal epithelium, their ability to exert similar regulation to that described in other somatic systems remains to be verified. Alternatively, recent work in our own laboratory has focused on the characterization of the temporal and spatial expression of the dynamin family of mechanoenzymes in the mouse epididymis (79). This family of proteins is of potential interest owing to their ability to couple both exo- and endocytotic processes. Indeed, while dynamin has been best studied in the context of clathrin-coated endocytosis from the plasma membrane, it is also implicated in formation and budding of transport vesicles from the Golgi network (80, 81), vesicle trafficking (82), orchestrating exocytotic events (83, 84), and in the regulation of microtubular and actin cytoskeletal dynamics (84, 85). Moreover, dynamin also has the potential to fine-tune exocytotic events by virtue of its ability to control the rate of fusion pore expansion, and thus the amount of cargo released from an exocytotic vesicle. In our analysis we found that, the dynamin 2 isoform is positioned within the vicinity of the Golgi apparatus of principal cells of the caput epididymis. Further, pharmacological inhibition of dynamin 2 selectively compromised the profile of proteins secreted from an immortalized caput epididymal cell line (79). On the basis of these data, we infer that dynamin 2 may contribute to the regulation of merocrine secretion by the mouse caput epithelium.

In addition to participating in merocrine secretion, there is also compelling evidence that the epididymal epithelium is heavily reliant on apocrine secretory pathways. Notably, apocrine secretion appears to underpin the transportation and release of epididymosomes into the epididymal lumen (Figure 2). This pathway, in turn, provides a mechanism for the release of proteins lacking an ER signal peptide and/or containing a glycosyl-phosphatidylinositol (GPI) anchor (86, 87); neither of which could be delivered to the epididymal luminal environment *via* the merocrine secretory pathway. As documented above, the epididymosomes released *via* apocrine secretion also contain a comprehensive profile of miRNAs and other sncRNAs, that themselves display marked segment specific differences (70). During intracellular transfer, the small epididymosome vesicles are first sequestered into large bleb-like structures that protrude from the apical margin of principal cells. The apical blebs eventually detach and disintegrate to release their encapsulated cargo within the lumen (11, 88). At present, the precise mechanisms by which the blebs are formed and detached remain to be determined. However, performed under conditions of stringent fixation, ultrastructural electron microscopy has revealed that the attachment of the apical blebs progressively narrows to form a stalk-like process that eventually undergoes scission to release the bleb into the lumen (88). This process appears to involve reorganization of cytoskeletal proteins such as myosin and β-actin (46, 89). In any case, the release of the epididymosomes into the luminal environment ideally positions this heterogeneous vesicle population to interact with the maturing spermatozoa.

### Epithelial Absorptive Pathways

The epithelial cells lining the initial segment of the mature mammalian epididymis have been shown to be very active in the uptake and recycling of the testicular contributions that enter the tract (3). One putative pathway for this absorption has been alluded to on the basis of apolipoprotein (apo) E receptor-2 (APOER2) expression in the principal cells of the initial segment. In this position, the APOER2 receptor is responsible for the clearance of clusterin, a glycoprotein implicated in lipid transport from spermatozoa to the principal cells. Accordingly, the inhibition of APOER2 leads to the accumulation of clusterin in the epididymal fluid (90). Additional luminal components such as androgen binding protein, transferrin, and alpha2-macroglobulin also appear to be recycled following selective adhesion to receptors located in the adluminal domain of principal cells; a portion of which are located in the initial segment, while others present with more diffuse localization throughout the downstream segments of the epididymis. While a portion of these proteins appear destined for disposal (91–93), others such as androgen binding protein have proven to be indispensable for the normal functioning of the epididymal principal cells (91). The balance of evidence indicates that, in contrast to the initial segment, the bulk of the absorptive and recycling activity of the distal epididymal regions (and in particular the cauda epididymis) resides in clear cells. This is certainly the case for immobilin, a large glycoprotein that is responsible for the creating of the viscoelastic luminal environment that serves to mechanically immobilize spermatozoa (94, 95). Immobilin is predominantly secreted into the proximal caput epididymis prior to the acquisition of the potential for sperm motility. Thereafter, immobilin forms an intimate association with the maturing sperm cells and physically restricts the propagation of a flagellar beat. In contrast, the principal cells of more distal segments secrete minimal immobilin, while the corresponding population of clear cells begins the task of absorbing excess immobilin (95). Differing patterns of absorption have been documented for alternative proteins such as that designated as epididymis-specific Inactive ribonuclease-like protein 10 (Protein Train A) (96); a protein that is secreted into the anterior segment of the bull epididymis *via* a classical merocrine pathway. Thereafter, Train A experiences a rapid reabsorption, such that this protein is unable to be detected in the epididymal lumen immediately adjacent to its site of secretion (96).

Receptor-mediated absorption is also involved in the recycling of the epididymal luminal contents (Figure 2). In this context, clusterin again serves as an interesting example. Indeed, in addition to the clusterin isoform that originates in the testes (and is subsequently absorbed by principal cells of the initial segment), an alternative isoform is abundantly secreted into the lumen of the proximal epididymis, whereupon it has been implicated in sperm maturation. Both *in vivo* and *in vitro* studies have revealed that epididymal sourced clusterin is recycled by downstream principal cells *via* interaction with low density lipoprotein receptor (LRP-2) (97). Accordingly, clusterin and LRP-2 are both found in association with the apical surface, coated pits, endocytic vesicles, and early endosomes of principal cells. Subsequently, only clusterin is detected in late endosomes and lysosomes, suggesting that LRP-2 is recycled back to the apical surface while clusterin is delivered to the lysosomes for degradation (97). This process can be prevented by presenting the cells with an excess of protein substrates that competitively bind to LRP-2. Such receptor-dependent recycling of luminal components is a commonly encountered phenomenon within the epididymis, with additional examples including transferrin and α2-macroglobulin (93). Epididymal epithelial cells also possess the ability to monitor the luminal environment and adjust their absorptive ability accordingly. In this context, the estrogen receptor α (ERα) has been identified as key sensor involved in the regulation of fluid reabsorption in the efferent ducts and initial segment of the epididymis. This system is apparently fine-tuned by a ubiquitin-dependent proteasome pathway that affords precise control over ERα turnover and degradation (98). Another interesting example of this phenomenon has been afforded by the analysis of an MFGE8 (formerly known as SED1) knockout mouse model (Table 1). The epididymal epithelial cells of these mice are characterized by increased accumulation of intracellular vesicles and an apical distribution of VATPase. Such changes are, in turn, reflected in epididymal luminal environment, which displays abnormal osmolarity (i.e., hypo-osmotic) and alkalinity; suggestive of the existence of a positive feedback loop responsible for regulating the behavior of the epithelial cells in response to changes in the luminal environment (99).

### Membrane Trafficking Machinery Involved in the Regulation of the Epididymal Environment

Despite recognition of the importance of bidirectional epithelial transport in regulating the epididymal luminal environment, little is currently known about the molecular machinery that controls these complementary pathways. Key elements are likely to include Soluble NSF Attachment Protein Receptor (SNARE) proteins, which have well-described roles in the regulation of membrane fusion activity in alternative tissue models. This activity requires the complementary action of different SNARE proteins contributed by vesicles (v-SNARE proteins) and target (t-SNARE proteins) membranes. Initiated by nucleation of the SNARE complex in response to calcium fluxes, the two opposing membranes are brought into close apposition and are thereafter able to engage in membrane fusion (100). The first evidence implicating SNARE proteins in the regulation of membrane fusion events in the epididymis arose from studies of the clear cell population (101). Functional analysis of these cells revealed that cellubrevin, a v-SNARE, is essential for acidification of the luminal environment. Accordingly, tetanus toxin-mediated cleavage of cellubrevin is able to inhibit proton secretion into the lumen (101). In addition to the lining epithelial cells, it is known that spermatozoa also harbor several t-SNARE and their cognate v-SNARE counterparts, which are localized to the plasma membrane and underlying outer acrosomal membranes, respectively. Such a location accords with the proposed role of SNARE proteins in regulating the membrane fusion events that underpin sperm acrosomal exocytosis (102–104). Notably however, this also ideally positions SNAREs to participate in the tethering of epididymosomes to the sperm surface and thereby facilitate the transfer of their cargo to the maturing cells. In agreement with this model, the requisite SNARE proteins necessary for constructing a functional membrane fusion complex have also been documented in epididymosomes (53, 101, 102). Nevertheless, there is currently limited experimental evidence to support the role of SNARE complexes in the regulation of sperm maturation.

Aside from SNARE proteins, recent proteomic analyses of luminal fluids obtained from the bovine epididymis have revealed the presence of at least 13 proteins implicated in clathrin-mediated endocytosis. Since this form of endocytosis is unlikely to occur in spermatozoa, these proteins may instead contribute the recycling of epididymal components *via* uptake into the surrounding epithelial cells (105). In this context, alternative regulators of membrane trafficking belonging to the Rab superfamily of GTPases have also been documented within epididymal luminal fluid, and more specifically, as part of the proteome of both human and bull epididymosomes (53, 106). Such findings are of interest as the Rab superfamily, which consists of some 30 members, have been implicated in regulating various membrane trafficking events including vesicle formation, sorting, release, and cargo transfer to recipient cells (100, 107). It is therefore tempting to speculate that Rab proteins may participate in epididymosome–sperm interaction/cargo transfer.

An additional family of membrane-trafficking proteins that warrant further investigation in the context of regulating epididymal function is that of the dynamin family of large GTPases. Indeed, our recent studies have shown that canonical dynamin isoforms display both cell, and segment-specific, differences in their profile of expression in the mouse epididymis. Specifically, dynamin 1 and 3 are mainly expressed in the corpus and cauda segments where they localize within the clear cell and principal cell populations, respectively. Of note, the differential localization of these dynamin isoforms contrasts the overlapping and redundant roles they display in neuronal tissues (108), suggesting that they may fulfill discrete functions in the epididymal tubule. Moreover, dynamin 1 was shown to be delivered to human spermatozoa during epididymal transit, in a mechanism that may involve epididymosomes (109). This compares favorably with the mouse in which caput and cauda spermatozoa display different patterns of dynamin 1 labeling (79). At present however, it remains to be determined whether dynamin 1 forms part of the cargo transferred between epididymosomes and maturing spermatozoa, or alternatively if it is instead involved in the regulation of epididymosome docking/fusion with spermatozoa. In contrast to dynamin isoforms 1 and 3, dynamin 2 localizes strongly to the Golgi apparatus in principal cells of the proximal epididymal segments, consistent with a role in mediating post-Golgi vesicle trafficking in the most active secretory cells of the tract. In more distal segments, dynamin 2 is detected in the microvilli and apical blebs lining the luminal border, suggesting it may be participate in the regulation of apocrine secretion (79).

### Epididymosome-Mediated Sperm-Soma Intracellular Communication

Among the varied molecular mechanisms that exert influence over the unique intraluminal environment of the epididymis, vesicle-dependent pathways of intercellular communication have emerged as being of fundamental importance. An obvious advantage afforded by the production of epididymosomes is the prospect of delivering macromolecular cargo to spermatozoa *en masse*. Additionally, the encapsulation of such cargo within a relatively stable membrane-bound structure could afford stability and protection against the potentially deleterious extracellular environment of the epididymal lumen. It is therefore perhaps not surprising that, in addition to their protein cargo, epididymosomes also comprise a heterogeneous population of small non-coding RNAs (sncRNA) including miRNA and tRNA fragments (70, 72). Like that of their protein cargo, these sncRNAs are available for direct transfer to the maturing spermatozoa. Accordingly, emerging work has shown that exposure of male mice to dietary perturbations (e.g., low protein diets) can markedly influence the sncRNA profiles of detected in the epididymis of these animals. Moreover, these changes are subsequently manifest in altered sperm sncRNA profiles, with epididymosomes having been implicated as the vector for delivery of this cargo to the maturing epididymal spermatozoa. Of some concern is the recognition that spermatozoa are subsequently able to relay these sncRNA to the oocyte during fertilization, whereupon they exert epigenetic control over early embryo development through targeting of a specific subset of genes (72). Such findings encourage a deeper understanding of the mechanisms underpinning the selective packaging of epididymosome cargo, the way in which this cargo is delivered to recipient cells (i.e., maturing spermatozoa and/or downstream epithelial cells) and the degree to which these vectors regulate the acquisition of functional competence in maturing spermatozoa.

This field of research is somewhat confounded by the fact that epididymosomes represent a heterogeneous population of vesicles. In this context, it has been shown that bovine epididymosomes collected from different epididymal segments are capable of transferring differing protein repertories to spermatozoa. Further, an excess of one population of epididymosomes (collected from caput segment) does not overtly influence the transfer efficacy of the alternative population (collected from cauda segment) during simultaneous co-incubation with spermatozoa (49). As an extension of this work, it has also recently been shown that epididymosomes can be subdivided into two discrete subpopulations owing to their size and molecular composition. The smaller of these populations measure ~10–100 nm in diameter and are distinguished based on the presence of tetraspanin-enriched microdomains containing both CD9 and its cooperative partner CD26. These epididymosomes also contain a relatively high concentration of proteins such as MIF and P25b, and display a preference to interact with live spermatozoa. These collective properties implicate this sub-class of epididymosomes in sperm maturation (110). The alternative subpopulation is characterized by the presence of epididymal sperm binding protein 1, and also by their propensity to interact with dead spermatozoa (52, 111). The overall heterogeneity of epididymosomes and their capacity to selectively interact with different sub-populations of spermatozoa suggests these interactions may be tightly regulated.

At present, however, the molecular mechanisms underpinning the biogenesis of different populations of epididymosomes, as well as those responsible for their interaction with spermatozoa remain to be established. Recent work has suggested the latter process may be initiated *via* the docking of GPI anchor(s) to the outer leaflet of the sperm surface lipid bilayer (87). Such docking is putatively followed by membrane fusion between the epididymosome and sperm membrane. In this regard, the adhesive/fusion properties of CD9 have identified this tetraspanin as a likely candidate in regulation of this fusion event (112, 113). Such a model is commensurate with the demonstration that anti-CD9 masking antibodies are able to reduce the efficacy of protein transfer from epididymosomes to spermatozoa (110). Notably, however, CD9 is unlikely to be the sole candidate since epididymosome–sperm interaction is characterized by a degree of selectivity in terms of the relayed content and their ability to discriminate between different populations of spermatozoa. As mentioned above, this is particularly the case in the bovine model where a subset of epididymosomes has been identified that do not possess CD9 and yet are still able to interact with spermatozoa (52). Owing to their role in receptor sequestration, lipid rafts have also been recently proposed as a “platform” to facilitate docking of the epididymosome and sperm membranes. Highly enriched in cholesterol and sphingolipid (114), these microdomains also compartmentalize GPI-anchored proteins such as P25b and SPAM1 (87) that themselves have been implicated in epididymosome-sperm adhesion. Thus, the release of P25b and SPAM1 proteins from sperm lipid rafts, *via* trypsin/pronase proteolysis, leads to a significant reduction in the efficacy of epididymosome cargo transfer to the sperm cells (115). However, it remains to be determined if the disruption of lipid raft integrity (e.g., through cholesterol sequestration using methyl-β-cyclodextrin) also compromises the docking of epididymosomes to the sperm surface.

Adding to this controversy is the suggestion that the interaction between epididymosomes and spermatozoa may not involve a complete fusion of their respective membranes (116). Rather, epididymosome adherence may be followed by creation of a transient fusion pore and subsequent release of the epididymosome once delivery of their cargo is complete. Accordingly, proteomic analyses of epididymosomes, and spermatozoa themselves, have identified a myriad of complementary trafficking proteins (e.g., SNARE proteins, Ras-like proteins, and dynamins) (53) that could regulate this form of intercellular communication. An intriguing aspect of this “kiss and run” model is that it could potentially facilitate bi-directional exchange of proteins and other macromolecules both into, and out of, the maturing sperm cell. It may also account for why a portion of epididymosomes persist in seminal fluids rather than being completely absorbed by spermatozoa within the duct. To the best of our knowledge, however, there is presently limited functional evidence linking any of the abovementioned trafficking proteins to a role in sperm-epididymosome interaction, and no evidence that epididymosomes can sequester proteins from spermatozoa. Irrespective, it has been shown that lipid labeled (Dilc12) epididymosomes originating from the median caput segment are able to be incorporated into distal caput epithelial cells *in vitro* (33). Such incorporation is time-dependent, with fluorescence imaging revealing a punctate distribution of Dilc12 within the epithelial cells after epididymosome interaction. This pattern of labeling is reminiscent of that observed after exosome interaction with somatic cells in other tissue models (117), thus indicating that sperm are not the sole recipients of epididymosome cargo.

## Conclusion

The epididymal milieu is undoubtedly crucial for promoting sperm maturation as well as supporting their storage. Indeed, since sperm are transcriptionally and translationally silent cells, their functional transformation relies entirely on the creation and maintenance of a highly specialized epididymal luminal milieu. The establishment of this unique epididymal microenvironment features the varied endocytotic and exocytotic contributions of the epithelial cells that line the duct. Unfortunately, our understanding of the molecular machinery the epididymal epithelial cells employ to facilitate these processes remains incomplete, as does our knowledge of how these cells are precisely regulated in different segments. Resolving these questions promises to inform our understanding of male fertility regulation with implications for contraceptive intervention and infertility diagnostics.

## Author Contributions

All authors made substantial contributions to the conception of this review and the critical appraisal of the literature summarized herein. WZ generated the initial draft of the manuscript, and this was critically revised by BN, GD, and MD. All authors approved the final version and submission of this article.

## Conflict of Interest Statement

The authors declare that this manuscript was prepared in the absence of any commercial or financial relationships or activities that could be construed as a potential conflict of interest.
